# Piezoelectric Materials Synthesized by the Hydrothermal Method and Their Applications

**DOI:** 10.3390/ma3125236

**Published:** 2010-12-09

**Authors:** Takeshi Morita

**Affiliations:** Graduate School of Frontier Sciences, The University of Tokyo, 5-1-5 Kashiwanoha, Kashiwa 277-8563 Japan; E-Mail: morita@k.u-tokyo.ac.jp; Tel.: +81-4-7136-4613; Fax: +81-4-7136-4619.

**Keywords:** hydrothermal method, PZT thin film, lead titanate, epitaxial film, lead-free piezoelectric ceramics

## Abstract

Synthesis by the hydrothermal method has various advantages, including low reaction temperature, three-dimensional substrate availability, and automatic polarization alignment during the process. In this review, powder synthesis, the fabrication of piezoelectric thin films, and their applications are introduced. A polycrystalline lead zirconate titanate (PZT) thin film was applied to a micro ultrasonic motor, and an epitaxial lead titanate (PbTiO_3_) thin film was estimated as a ferroelectric data storage medium. Ferroelectric and piezoelectric properties were successfully obtained for epitaxial PbTiO_3_ films. As lead-free piezoelectric powders, KNbO_3_ and NaNbO_3_ powders were synthesized by the hydrothermal method and sintered together to form (K,Na)NbO_3_ ceramics, from which reasonable piezoelectric performance was achieved.

## 1. Introduction

Piezoelectric materials are widely utilized as electromechanical energy converters for actuators, sensors, and transformers [1,2]. Compared to magnetic devices, piezoelectric devices have a simple structure and high energy density, which contributes to the distinguished performance observed for miniaturized systems. Therefore, suitable deposition processes are necessary to fabricate piezoelectric films in such micro actuators and sensors.

Among the various deposition processes, the hydrothermal method has a unique advantage of low reaction temperature, less than 200 °C [3,4,5,6,7,8,9,10,11,12] in the case of lead zirconate titanate (PZT) or lead titanate (PbTiO_3_) films, which is below the Curie temperature. This low reaction temperature results in excellent crystal quality by reducing the residual strain. Furthermore, direct synthesis as an ionic reaction in solution enables high-quality thin film deposition without impurities. Other advantages of PZT or PbTiO_3_ films synthesized via the hydrothermal method are large thickness, the availability of a three dimensional structure substrate and self-alignment polarization.

In addition to PZT thin films, environmental friendly piezoelectric materials, such as lead-free piezoelectric powders synthesized by the hydrothermal method, have been investigated [13,14,15]. One of the promising lead-free piezoelectric materials is potassium niobate-based ceramics, such as (K,Na)NbO_3_. Synthesis of such materials by the conventional solid state process has some serious problems, due to the instability and deliquescence of potassium carbonate, which is generally used as a potassium source. To overcome this problem, powders produced by the hydrothermal method were examined, and good piezoelectric performance of the sintered ceramics was successfully achieved. 

In this review paper, the hydrothermal synthesis method is explained for ferroelectric materials, and a micro ultrasonic motor [16,17] is introduced as an example of a polycrystalline PZT thin film application. A hydrothermally deposited epitaxial PbTiO_3_ thin film [7] is also demonstrated as a memory medium for a ferroelectric hard-disk device. In addition, the hydrothermal fabrication process for lead-free piezoelectric powders, KNbO_3_ and NaNbO_3_, is described, and the piezoelectric performance of a sintered solid-phase (K,Na)NbO_3_ of these powders is presented [13,14,15].

## 2. Hydrothermal Synthesis of PZT or PbTiO_3_ Films

**Figure 1:**
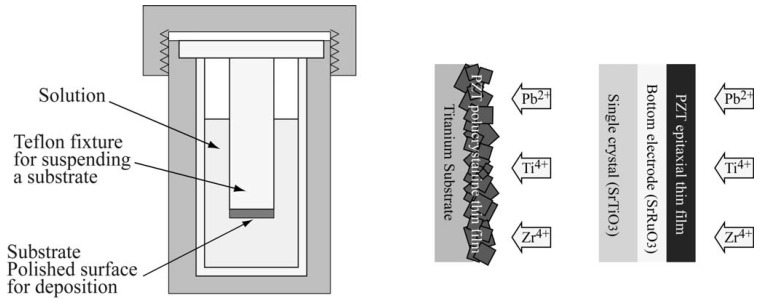
Hydrothermal method to deposit polycrystalline and epitaxial PZT.

The hydrothermal method is a unique method to obtain piezoelectric materials by utilizing chemical reaction in solution at a reaction temperature of less than 200 °C (Figure 1). In case of PZT thin film deposition, the ion sources, Pb(NO_3_)_2_, ZrOCl_2_·8H_2_O, and TiO_2_ or TiCl_4_, are placed in a high pressure container with KOH solution. A substrate is then placed into the solution, and the high pressure container is placed in an oven for a predetermined time, for example, 24 h. Thicker films can be realized by repeating the reaction process. Detailed reaction conditions are given in [4,5,6,7,8].

Polycrystalline PZT has been obtained on a titanium metal substrate [4,5]. With respect to ease of fabrication, a metal substrate has advantages over a single crystal substrate. However, high-quality epitaxial PZT or PbTiO_3_ thin films can be deposited on a single crystal substrate such as SrTiO_3_ [6,7,8,9,10]. SrTiO_3_ has superior lattice matching to PZT or PbTiO_3_ and has high resistance against strongly alkali conditions. By improving the reaction conditions and adapting the SrRuO_3_ bottom electrode on SrTiO_3_, the ferroelectric and the piezoelectric properties were successfully measured [8]. These results confirmed that high-quality piezoelectric film can be deposited by the hydrothermal method.

### 2.1. Micro Ultrasonic Motor Using Polycrystalline PZT Thin Film

**Figure 2:**
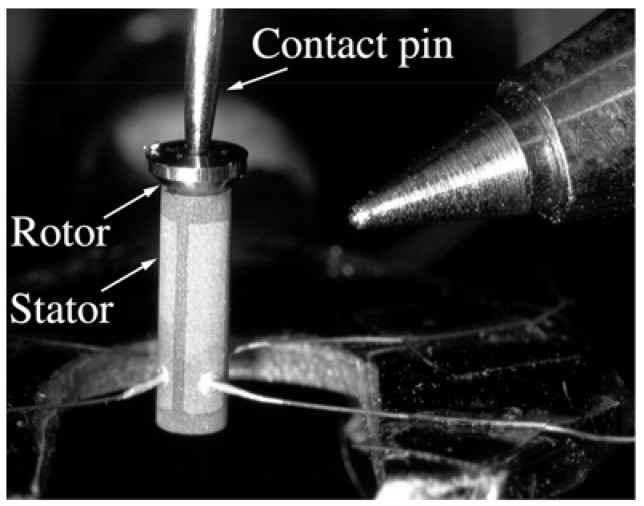
Cylindrical-shaped micro ultrasonic motor using polycrystalline PZT film [17].

**Figure 3:**
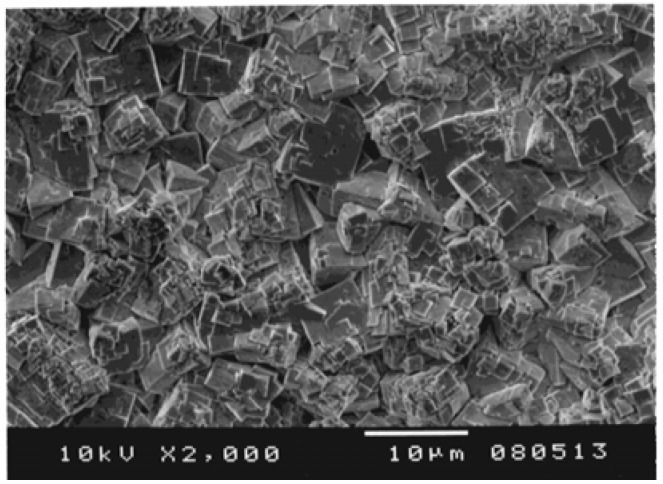
Polycrystalline PZT thin film deposited by the hydrothermal method.

As an example of the application of polycrystalline PZT thin films, a micro ultrasonic motor was fabricated and is shown in Figure 2 [16,17]. The stator transducer has an outer diameter of 1.4 mm, an inner diameter of 1.2 mm, and is 5 mm long. The base metal of the stator transducer was titanium, and a PZT thin film was deposited on the sidewall, as shown in Figure 3, using an improved hydrothermal method. The thickness of the PZT thin film was increased to 12 μm by repeating the hydrothermal deposition reaction four times. The film thickness saturates during the hydrothermal reaction, and by repeating the reaction times, it could be increased. The poling direction was automatically aligned to the thickness direction. Four gold electrodes were deposited onto the PZT thin film by evaporation of gold with a metal mask.

The ultrasonic motor is a mode-rotation type, which utilizes the first bending vibration mode. With four driving electrical sources connected to each electrode, the bending vibration is degenerated, with phase differences of 90° to each other. The traveling wave is propagated at both end surfaces of the stator, and the rotor loaded on the transducer rotates by frictional force. The driving direction is reversible by changing the electrical phase shift from 90 to −90°.

The maximum torque was 0.67 μNm with 5.3 mN pre-load and 20 V input voltage. The driving frequency was 227 kHz, which corresponded to the resonant frequency of the transducer. Under the same conditions, the convergent revolution speed was 680 rpm. From the vibration performance obtained with three different dimension stators, the piezoelectric coefficient *e_31_* was estimated to be −0.57 C/m^2^. Smaller ultrasonic motor performance was estimated from this value. The output torque of 27 nNm for a 100 μm diameter motor is sufficient as a micro actuator when compared with an electrostatic micro motor or a previous disk type micro ultrasonic motor, of which the output torque was in the order of pNm. Thus, the output torque indicates that the ultrasonic motor is a promising actuator for micromechanical systems. A tactile sensor [18] and miniature earphone [19] have also been reported as applications using polycrystalline PZT films synthesized by the hydrothermal method.

### 2.2. Piezoelectric Properties of Epitaxial PbTiO_3_ Thin Film

PbTiO_3_ is a fundamental ferroelectric material and is important as a component of solid solutions such as PZT and lead magnesium niobate-lead titanate (PMN-PT). However, it has been difficult to realize PbTiO_3_ with sufficient resistivity, and there have been few experimental results regarding the ferroelectric and piezoelectric properties of PbTiO_3_.

**Figure 4:**
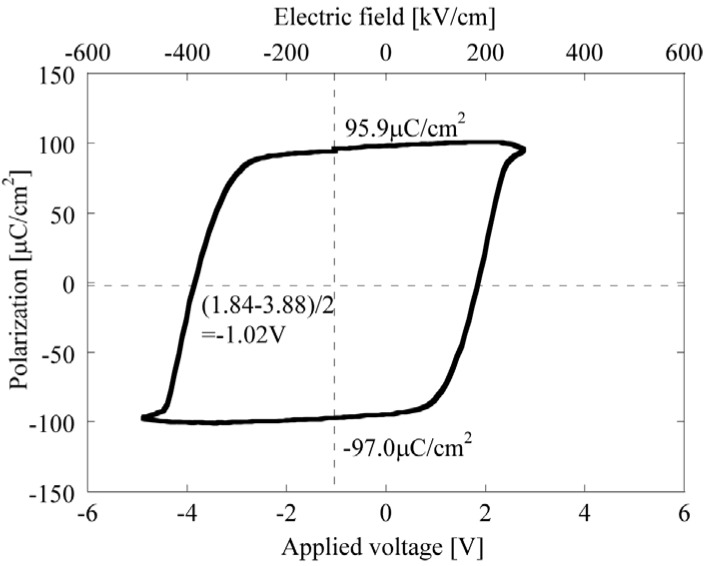
Ferroelectric hysteresis curve of the PbTiO_3_ thin film [7].

**Figure 5:**
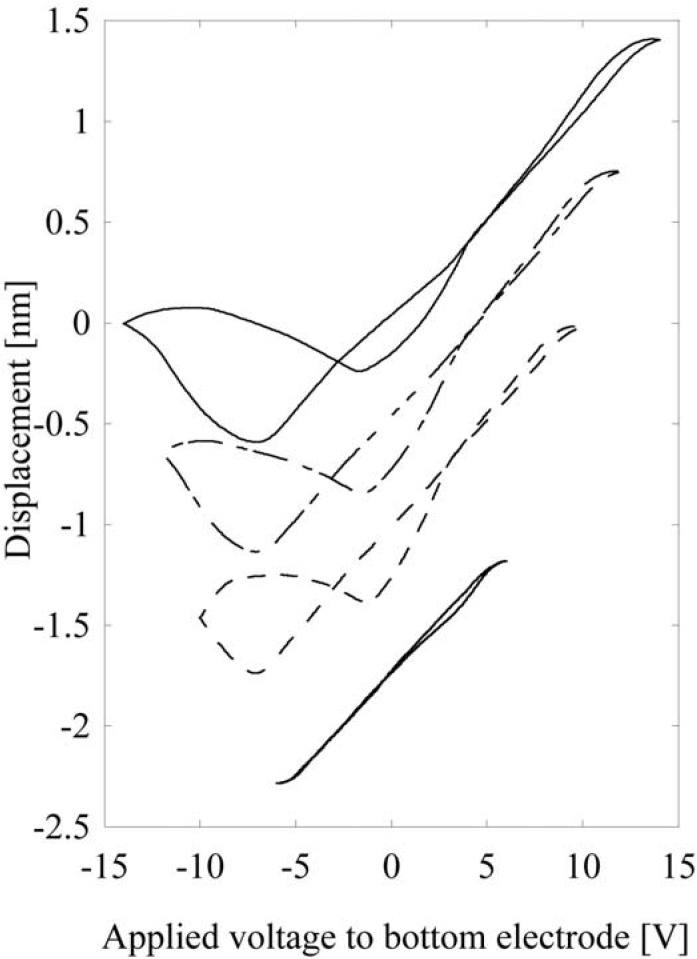
Relationship between piezoelectric displacement and input voltage [8].

A high-quality crystal PbTiO_3_ epitaxial thin film was successfully obtained by the hydrothermal method [7,8]. After a 24 h reaction, the thickness was 100 nm. This film indicated 96.5 μC/cm^2^ remnant polarization in DE hysteresis measurements as shown in Figure 4. The deposited film was perfectly epitaxial with a polarization direction from the top surface to the underlying SrRuO_3_ electrode without poling treatment. A thinner 50 nm epitaxial thin film was examined as a ferroelectric medium for an ultra-high-density storage system. This system utilized a scanning nonlinear dielectric microscope (SNDM) invented by Cho *et al.* [20,21]. A nanodot pattern was obtained in this system, as shown in Figure 5, which suggests that hydrothermal PbTiO_3_ thin film can be utilized as data media for ferroelectric data storage systems [21].

**Figure 6:**
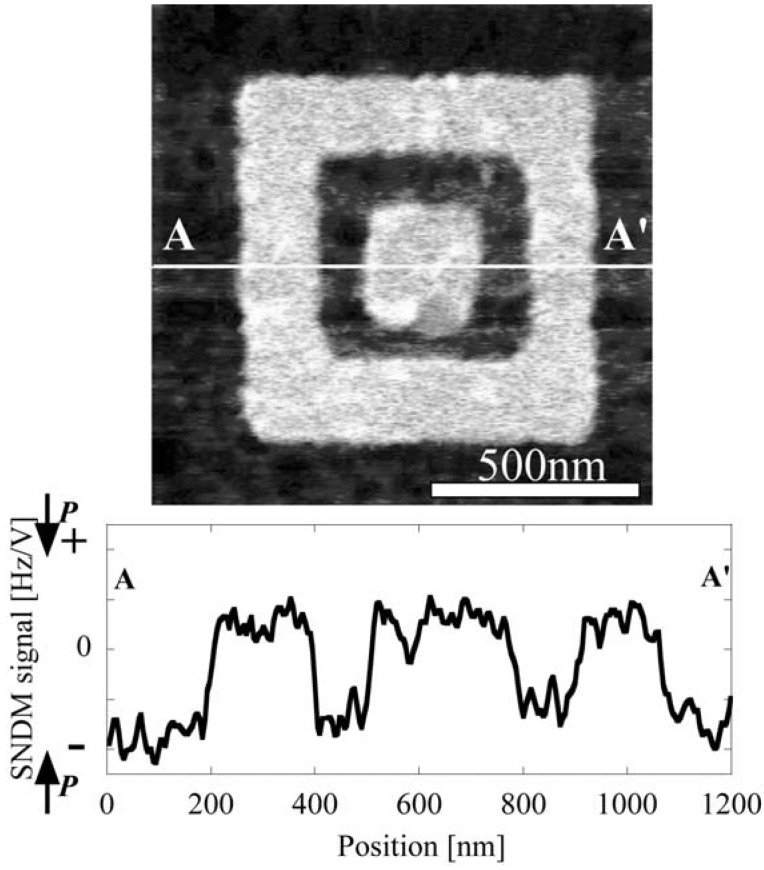
Domain patterning in the PbTiO_3_ epitaxial thin film using a scanning nonlinear dielectric microscope (SNDM) [8].

Besides application in ultra-high density data storage media, PbTiO_3_ films have significant potential for use as piezoelectric actuators and sensors, particularly as high frequency ultrasonic transducers. By changing the reaction parameters, 430 nm thick PbTiO_3_ epitaxial film was deposited and its piezoelectric properties were examined. The domain structure was composed of dominant +c-domain and a small amount of a-domain. The strain has splendid linearity, as shown in Figure 6, and the *d_33eff_* coefficient was found to be 97 pC/N, which is larger than the predicted value [22]. The large piezoelectric performance and linearity are especially suitable for ultrasonic transducer applications.

## 3. Lead-free Piezoelectric Ceramics from Hydrothermal Powders

Lead-free piezoelectric ceramics have been widely studied as replacements for PZT ceramics. Alkaline niobate-based piezoelectric ceramics have good piezoelectric properties and high Curie temperatures. Among these, (K,Na)NbO_3_ is considered as a promising candidate for lead-free piezoelectric ceramics. Usually, the solid solution method is used to obtain source powders; however, potassium carbonate (K_2_CO_3_) as a potassium source is unstable and quite difficult to weigh due to its deliquescence. Another problem is that the potassium is easily evaporated during the sintering process.

In contrast, the hydrothermal method is proposed to obtain source powders for these ceramics, and it has been verified that this method enables the production of high-quality powders [13,14]. Crystallization from the solution was achieved with the hydrothermal method, so that pure crystal powder could be obtained without difficulty. In addition, the potassium to niobium ratio was automatically controlled to be one. Therefore, the simple process and low reaction temperature (around 200 °C) provide this method with certain advantages over other conventional methods. 

**Figure 7:**
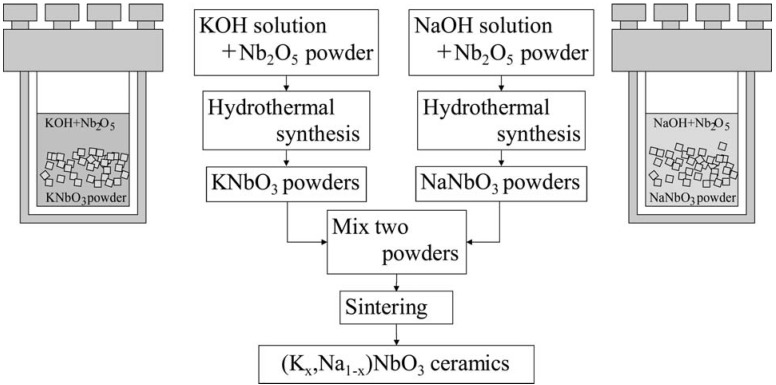
Hydrothermal method to obtain lead-free piezoelectric powders and the sintering process.

**Figure 8:**
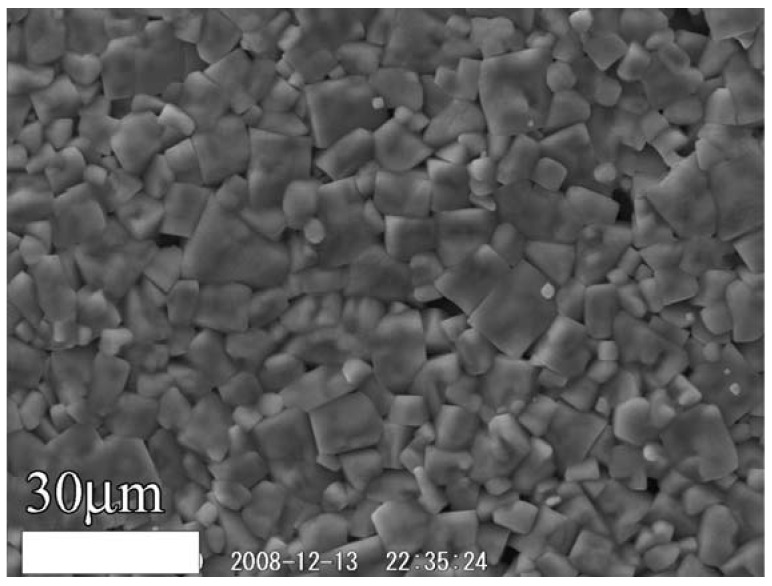
SEM photograph of (K,Na)NbO_3_ ceramic sintered from powders synthesized by the hydrothermal method [14].

**Figure 9:**
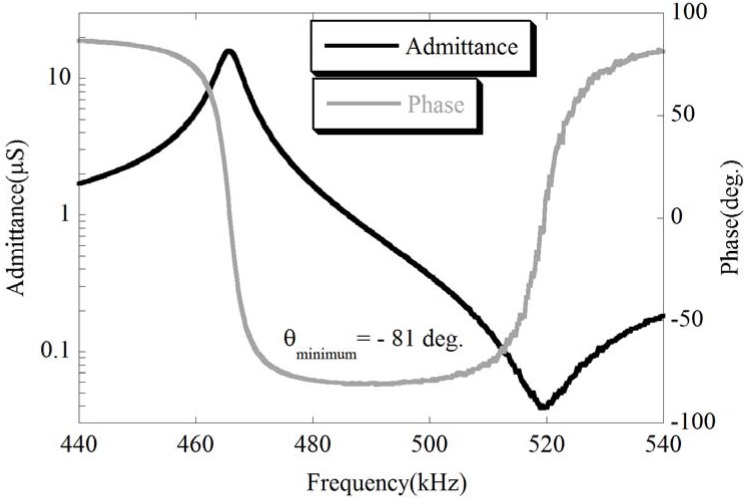
Admittance and phase curve of the (K,Na)NbO_3_ ceramic [14].

The KNbO_3_ and NaNbO_3_ powders were sintered to form the (K,Na)NbO_3_ solid solution, as shown in Figure 7. An SEM micrograph of the sintered (K,Na)NbO_3_ ceramics is shown in Figure 8. The piezoelectric performance of (K,Na)NbO_3_ was estimated from admittance measurements, one example of which is shown in Figure 9. Properties obtained for the sintered (K,Na)NbO_3_ ceramic were the electromechanical coupling factors *k_p_* (0.32) and *k_33_* (0.48), the mechanical quality factor Qm 71 (radial mode), 118 ((33)mode), and the piezoelectric constant *d_33_* (107 pC/N) [14].

## 4. Summary

The hydrothermal method was introduced as a simple, low-temperature process for the deposition of PZT polycrystalline films and epitaxial PbTiO_3_ film. A micro ultrasonic motor was fabricated and successfully operated as an application of polycrystalline PZT thin film. *d_33_* measurements were carried out for the PbTiO_3_ epitaxial film, and the results indicated the potential of the hydrothermal method for the fabrication of ultra-high-quality films. KNbO_3_ and NaNbO_3_ powders were synthesized as source powders for the synthesis of lead-free piezoelectric ceramics, and they were sintered together to form a (K,Na)NbO_3_ solid solution, the piezoelectric properties of which indicated that the hydrothermal method has significant advantages for the fabrication of lead-free piezoelectric materials.
